# Understanding the effectiveness and underlying mechanisms of lifestyle modification interventions in adults with learning disabilities: protocol for a mixed-methods systematic review

**DOI:** 10.1186/s13643-021-01808-0

**Published:** 2021-09-20

**Authors:** Dikshyanta Rana, Sophie Westrop, Evi Germeni, Arlene McGarty, Louisa Ells, Phillippa Lally, Michael McEwan, Craig Melville, Leanne Harris, Olivia Wu

**Affiliations:** 1 Institute of Health & Wellbeing, University of Glasgow, Glasgow, G12 8RZ UK; 2 School of Clinical and Applied Sciences, Leeds Beckett University, City Campus, Leeds, LS1 3HE UK; 3 UCL Institute of Epidemiology and Health Care, University College London, London, WC1E 6BT UK; 4 People First (Scotland), Edinburgh, EH7 5PW UK; 5 School of Medicine, Dentistry & Nursing, University of Glasgow, Glasgow, G31 2ER UK

**Keywords:** Systematic review, Network meta-analysis, Component network meta-analysis, Realist review, Logic model, Learning disability, Intellectual disability, Lifestyle interventions

## Abstract

**Background:**

Adults with learning disabilities have an increased disposition to unhealthy lifestyle behaviours which often occur simultaneously. Existing studies focus on complex interventions targeting unhealthy diet, physical inactivity, sedentary behaviour, smoking, and alcohol use to reduce health risks experienced. It is essential to understand how well these interventions work, what works, for whom, in what context and why. This study aims to investigate the effectiveness and underlying mechanisms of lifestyle modification interventions for adults with learning disabilities.

**Methods:**

This is a mixed-methods systematic review consisting of a network meta-analysis (NMA) and realist synthesis. Electronic databases (ASSIA, CINAHL, EMBASE, MEDLINE, and PsycINFO) will be searched from inception to 14 January 2021 with no language restriction. Additionally, trial registries, grey literature databases and references lists will be searched. Studies related to lifestyle modification interventions on the adult population (>18 years) with learning disabilities will be eligible for inclusion. Two independent researchers will screen studies, extract data and assess its quality and risk of bias using the Cochrane Collaboration’s Risk of Bias Assessment Tool (RoB Version 2) and ROBINS-I. The strength of the body of evidence will be assessed based on the GRADE approach. The NMA will incorporate results from RCTs and quasi-experimental studies to estimate the effectiveness of various lifestyle interventions. Where appropriate, a component NMA (CNMA) will be used to estimate effectiveness. The realist synthesis will complement and explain the findings of NMA and CNMA by including additional qualitative and mixed-methods studies. Studies will be included based on their relevance to the programme theory and the rigour of their methods, as determined by quality appraisal tools appropriate to the study design. Results from both syntheses will be incorporated into a logic model.

**Discussion:**

The paucity of population-specific lifestyle interventions contributes to the challenges of behaviour change in adults with learning disabilities. This study will provide an evidence-base from which various stakeholders can develop effective interventions for adults with learning disabilities. The evidence will also help prioritise and inform research recommendations for future primary research so that people with learning disabilities live happier, healthier and longer lives.

**Trial registration:**

PROSPERO CRD 42020223290

**Supplementary Information:**

The online version contains supplementary material available at 10.1186/s13643-021-01808-0.

## Background

The transition of care for adults with learning disabilities away from large institutions towards care in the community has led to an increased reliance on mainstream healthcare services. Consequently, reintegration into the community has exposed them to social and environmental pressures [1]. This is concerning as adults with learning disabilities engage in a cluster of health risk behaviours predominantly eating an unhealthy diet, physical inactivity, sedentary behaviour, smoking and alcohol use [2, 3]. Gateway theories propose that engaging in one health risk behaviour increases the likelihood of engaging in multiple health risk behaviours [4]. This increased disposition to unhealthy lifestyle behaviours, which rarely occurs in isolation, has an adverse impact on their health [5]. Adults with learning disabilities experience higher comorbidity rates and premature mortality compared to the general population [6, 7]. They have an increased risk of developing preventable diseases such as cardiovascular disease, obesity, type-2 diabetes and some cancers [8].

Research on the determinants of health supports the need to prioritise these health risk behaviours. Adults with learning disabilities have low fruit and vegetable consumption and higher fat intake. They spend a high proportion of their day sedentary [9, 10] and engage in low levels of health-enhancing physical activity [11]. These unhealthy lifestyle behaviours create an imbalance in energy intake and expenditure, contributing to a high prevalence of obesity in adults with learning disabilities compared to the general population [1, 3, 12]. Conflicting findings related to the prevalence of smoking and alcohol consumption in adults with learning disabilities exist as these rates appear to be similar to the general population [13, 14]. As a leading risk factor for the development of cardiovascular disease and cancer, smoking aggravates secondary conditions such as type 2 diabetes, which are highly prevalent among adults with learning disabilities [15]. Additionally, excessive alcohol consumption is associated with several concerns related to the risk of personal safety and interpersonal relationships in adults with learning disabilities due to their impaired judgement and risk-taking, and long-term physical and mental health issues [16]. Furthermore, adults with mild learning disabilities are more vulnerable to social and environmental pressures [16] than adults with severe learning disabilities and the general population. Compared to these two populations, they have higher rates of obesity, smoking, alcohol consumption and clustering of unhealthy lifestyle behaviours [3].

The latest review of multicomponent weight management interventions concluded that current interventions based on a health education approach are not effective in supporting a clinically meaningful weight loss (5–10% of initial body weight) [17]. It recommended weight management interventions adhere to clinical guidelines based on a daily energy deficit diet (EDD) of 600 kcal/ day to achieve a clinically meaningful effect [18, 19]. Only two studies considering an EDD has achieved sustainable, clinically meaningful weight loss till date [20, 21]. A systematic review of smoking and alcohol cessation interventions reported heterogeneity in study designs and intervention components, which precluded quantitative assessment of these interventions’ effectiveness [22]. Similarly, in another systematic review of physical activity and dietary interventions, effect sizes were not quantified due to high heterogeneity [23, 24]. A review and meta-analysis of interventions on multiple lifestyle behaviours (nutrition and physical activity) reported moderate effects on anthropometric and physical activity outcomes [25]. However, the effects were only statistically significant for waist circumference, and it was not reported whether these were clinically meaningful.

There are some more limitations to existing systematic reviews. The reviews focused primarily on data from randomised controlled trials (RCTs) of single behaviours or health outcomes [23, 24]. Thus, it did not consider that individual studies may report multiple effect sizes correlated with each other due to multiple outcome measures and/or the same outcome measures at multiple time points. Existing meta-analyses have limited comparisons between any lifestyle interventions versus control. Rather than lumping these interventions as a homogenous whole, it is critical to highlight that complex interventions are made up of diverse components instead and identify these effective components.

Therefore, our study will determine the effectiveness of lifestyle modification interventions and explain what works, for whom, in what circumstances and why. This is necessary to ensure interventions reflect the needs of adults with learning disabilities and effectively promote healthy lifestyles to improve overall health and wellbeing subsequently.

The aim of the review is to investigate the effectiveness and underlying mechanisms of lifestyle modification interventions in adults with learning disabilities. This mixed-methods study will be conducted to address the following objectives:

- To determine the effectiveness of different lifestyle modification interventions and the components within the interventions in adults with learning disabilities
- To establish how lifestyle modification interventions for adults with learning disabilities work, for whom they work, as well as why they may work in particular circumstances and not in others
- To integrate the findings of the quantitative and qualitative syntheses using a logic model
- To identify future research priorities to develop lifestyle modification interventions for the NHS and social care services to improve the health of adults with learning disabilities

## Methods

### Registration

This protocol is registered in the PROSPERO (ID: CRD 42020223290). The present protocol has been reported according to the Preferred Reporting Items for Systematic Reviews and Meta-Analyses Protocols (PRISMA-P) statement (see Additional file 1) [26].

### Data source and strategy

An extensive and comprehensive search of relevant studies will be performed through electronic databases. The following databases will be searched from inception to 14 January 2021: Applied Social Sciences Index and Abstracts (ASSIA); Cumulative Index to Nursing and Allied Health Literature (CINAHL); EMBASE; MEDLINE, and PsycINFO. Registered and ongoing clinical trials will be searched in these databases: Cochrane Central Register of Controlled Trials (CENTRAL); ClinicalTrials.gov; International Standard Randomised Controlled Trials Number (ISRCTN); and Evidence for Policy and Practice Information and Co-ordinating Centre (EPPI-Centre). Grey literature will be identified in Open Systems for Information on Grey Literature in Europe (OpenSIGLE) and Google Scholar. We will hand search reference lists of relevant systematic reviews and included studies.

The search strategy will be developed according to the PICOS framework defined by the review question domains which includes the health condition (learning disabilities), health risk behaviours (diet, physical inactivity, sedentary behaviour, smoking, alcohol) and interventions with desired outcomes. Existing systematic reviews and search words in papers within the related domains will be referred to develop a thorough search strategy. Alternative terms (e.g., exercise or lifestyle physical activity) will be used to maximise the identification of relevant papers. Search terms in truncated formats will permit more comprehensive terms and different sets of the terms to be searched for simultaneously. Appropriate Boolean operators will be used. The full search strategy will be adapted for each database, and include medical subject headings (MeSH) and free text words. A version of search strategy, limited to human population, in MEDLINE is available (see Additional file 2).

### Eligibility criteria

#### Population

We will include studies involving adults (age of 18 years and above) diagnosed with learning disabilities (or equivalent term, e.g., intellectual disabilities). To ensure consistency in the definition of learning disabilities across the studies, international definition which considers it a limitation in intellectual functioning (intelligence quotient < 70) and adaptive behaviour with onset before age 18 years will be followed. Each study will be judged on how appropriately adults with learning disabilities are defined, as criteria for its definition may differ across studies.

#### Interventions and comparators

The review will include all lifestyle behaviour change interventions developed to modify one or more of the following health risk behaviours: smoking (cigarettes or tobacco), alcohol consumption, diet, physical inactivity and sedentary behaviour. Interventions will be categorised on the basis of the lifestyle behaviour it targets. There will be no restrictions related to the intervention settings (e.g., community or domiciliary setting). These interventions will be compared against active comparators, or control interventions, or treatment as usual arms (standard care at the time that an eligible study was done), or post-study arms.

#### Outcomes

Our primary outcome of interest will be any outcome related to the effectiveness of lifestyle modification interventions. For example, outcomes that show changes in lifestyle behaviours smoking (e.g., number of cigarettes), alcohol use (e.g., number of units), diet (e.g., energy intake), physical activity (e.g., minutes per day and intensity) and sedentary behaviour (e.g., minutes per day).

We expect these effectiveness outcomes to be of varied nature, such as anthropometric (e.g., weight), behavioural (e.g., dietary intake), cardiorespiratory (e.g., aerobic fitness), metabolic (e.g., lipoprotein profiles), glycaemic control (e.g., blood glucose), functional (e.g., muscle strength), psychosocial (e.g., self-efficacy) and knowledge-related (e.g., alcohol-related knowledge).

Some potential secondary outcomes of interest include health-related quality of life/wellbeing, attrition rate and reasons for drop out, cost-effectiveness and adverse events.

#### Study design

The quantitative synthesis will include all comparative effectiveness studies (RCTs, quasi-experimental studies with control or comparator intervention, and uncontrolled pre-post design) involving lifestyle modification interventions in adults with learning disabilities. For the realist synthesis, we will use the studies included in the quantitative synthesis and published or unpublished studies of any methodological study designs (i.e., quantitative, qualitative and mixed-methods), based on relevance to developing a programme theory.

### Study selection and data extraction

Two reviewers will independently conduct the study selection, data extraction, and coding process. COVIDENCE software and EndNote X9 will be used to streamline this process. Article titles and abstracts will be screened against the inclusion criteria by the reviewers. Full-text articles that appear to meet the inclusion criteria or where there is any uncertainty will be reviewed. The results of the search strategy and selection process will be recorded in a flow chart. Studies will initially not be restricted by study design to identify papers that may be relevant for the realist synthesis. Papers that meet the criteria for the quantitative synthesis will then be identified. Relevant systematic reviews will be stored for further handsearching. We will record the excluded studies and reasons for exclusions of full-text articles.

Any disagreement between the reviewers over particular studies’ eligibility will be resolved through discussion with a third reviewer. Where multiple papers pertaining to the same study are identified (e.g., protocol paper and outcome paper or outcomes paper of the same study reporting on different follow-up time points), data will be extracted with care to avoid duplication of information. These studies will be linked together under one study identification number. We will contact the authors of papers included to request information on missing data or re-verify key characteristics such as aspects of an intervention, wherever appropriate.

Our data extraction form will be adapted from a previous review of lifestyle interventions [25] and the sample forms presented in the Cochrane Handbook [27]. It will be designed in Microsoft Excel. Our Patient and Public Involvement (PPI) representatives will be consulted to ensure that all important data are captured. Broadly, the following data will be extracted from the relevant studies based on lifestyle modification behaviours targeted, including multiple behaviours:

- General study characteristics: authors, year, country, funder, study design, unit of allocation (individual, cluster, group)
- Sample characteristics: sample size, level of disability, age, sex, ethnicity, living status (alone or with carer/family)
- Intervention and comparator characteristics: intervention and comparator detail, whether the intervention is theory-based and extent to which theory has been used, behaviour change techniques, behaviour targeted, delivery of intervention, setting, duration, frequency and intensity, definition of key parameters
- Outcomes: outcomes of interest, timepoints measured, follow-up period, attrition rates, intervention fidelity
- Data analysis and conclusions: method of analysis, key findings

The intervention components will be coded for whether the interventions were accessible for the population (e.g., provided easy read resources), whether behavioural recommendations were specified, whether the intervention is based on an explicit theory, and/or employed behaviour change techniques (BCTs). Based on the work by Michie et al. [28] the extent to which theory has been used in the intervention design will be coded using a 19-item Theory Coding Scheme (TCS). This includes whether a theory or model was mentioned, how theories were used in theory development, how intervention evaluations tested the theory and the implications of the results for future theory development. We will also code the intervention and comparator treatments using the 93-item BCT taxonomy [29]. BCT will only be coded if there is sufficient description matching the technique definition. It will aid us in identifying the active components of the interventions and comparator treatments.

### Quality and risk of bias assessment in studies included in the systematic review and network meta-analysis

For papers included in the systematic review and network meta-analysis, two reviewers will independently assess the quality and risk of bias of the included studies using validated and study-design appropriate tools for papers included in the quantitative data synthesis. The risk of bias for RCTs will be assessed by the Cochrane Collaboration’s Risk of Bias Assessment Tool (RoB Version 2) [30]. It constitutes six domains: selection bias (adequate sequence generation and allocation concealment); performance bias (blinding of participants); detection bias (blinding of outcome assessors); attrition bias (clear account of dropouts and exclusions); and reporting bias (selective outcome reporting). The risk of bias of non-randomised trials will be assessed using the Risk Of Bias In Non-randomised Studies - of Interventions (ROBINS-I) tool [31]. These specifically evaluate risk of bias applicable to case-control or cohort studies. All studies will be judged as high, low or unclear risk of bias.

### Confidence in cumulative evidence

The overall quality of included studies will be assessed per the Grading of Recommendations Assessment, Development and Evaluation (GRADE) methodology [32] to inform the strength of conclusion of the effectiveness of lifestyle modification interventions. The evaluation criteria include risk of bias, consistency (heterogeneity), directness (generalisability), precision (statistical significance of effect measures) and publication bias. The quality of evidence is graded as high, moderate, low or very low.

### Quantitative data synthesis

We plan to perform a network meta-analysis (NMA) to simultaneously compare multiple interventions. The analysis will follow the general principles set out by the International Society for Pharmacoeconomics Outcomes Research (ISPOR) Taskforce [33, 34].

Characteristics of included studies will be described. We will provide a descriptive summary of findings, including contextual factors such as participant characteristics, outcomes which lack sufficient data or studies which were inappropriate to combine statistically.

The NMA will be conducted at the intervention level and the component level. At the intervention level NMA, we will estimate the relative effectiveness of all interventions on individual outcomes using direct (from individual studies) and indirect evidence. Component network meta-analysis (CNMA) will be conducted according to the components-based approach developed by Welton and Freeman et al. [35, 36]. CNMA allows assessment of the effectiveness of interventions and the components that contribute to improvements in modifiable health risk factors in adults with learning disabilities. Here, each intervention’s effect will be dismantled by modelling component-specific effects to answer if interventions with a particular component or combinations of components are effective. We plan to classify the components based on theories and BCT codes, if appropriate. 

Our analysis will be conducted in R software following a Bayesian framework. Effect sizes of included comparisons will be expressed as standardised mean differences (SMD) if the outcome is continuous and odds ratio (OR) if the outcome is dichotomous. A network graph will be used to assess the geometry of the network. It will enable us to view the structure of comparisons in our network. Description of network geometry will depend on number of nodes i.e., interventions, edges which represent head-to-head (direct) comparison evidence and thickness of the edges which is proportional to the number of studies included.

Following the Bayesian framework, a minimally informative prior distribution for the overall effect size and the between-study heterogeneity will be specified. We anticipate statistical heterogeneity due to variation in factors such as participant characteristics, types of interventions and outcome measures. Therefore, we will use random effects model to pool effect sizes. The convergence of the model will be examined. We will report the posterior mean and median effect with their 95% credible intervals (CrI). Common between-study variance (Tau^2^) will also be reported. A large Tau^2^ means that there are important differences between the true effects. Deviance information criterion (DIC), which is equal to the sum of the residual deviance’s posterior mean and the effective number of parameters, will be used to assess the fit and parsimony of the model [37].

In case of CNMA, we will dismantle the effect of each intervention by modelling its components in three models [35, 36]. Following are the three meta-regression-based models that will be fitted, and its results reported along with 95% credible intervals:

- An additive main effects model: The model assumes that each intervention’s effect is the sum of the effects of the component parts. The model will aid us understand the effectiveness of an intervention containing certain specific components compared to interventions without the same components.
- An extended additive effects model: A two-factor interaction model which allows pairs of components to have either a larger (synergistic) or smaller (antagonistic) effect than would be expected from the sum of their effects alone. The model aids in understanding if interventions containing specific pairs of components are effective.
- A saturated CNMA model/full-interaction model: This model is the standard NMA model in which each of the different combinations of components is considered a distinct intervention with its own effect, regardless of whether it is made up of one or more components.

All interventions and components will be ranked to provide a probability of each intervention being considered the best in each outcome using methods like surface under the cumulative ranking (SUCRA) score.

The validity of NMA depends on two most important assumptions of transitivity and consistency. Transitivity means that the prevalence of effect modifiers is similar among studies [38]. It suggests that intervention A is similar when it appears in A versus B and A versus C studies [39]. It can be examined by comparing the distribution of potential effect modifiers across the different comparisons [40]. Therefore, we will create a table to facilitate an inspection of important trial and participant characteristics that we consider most likely to be effect modifiers. These include factors such as age, gender, level of learning disabilities, inclusion/exclusion criteria, intensity and mode of delivery of the interventions etc. Consistency refers to the equivalence of effects from direct and indirect evidence across the different comparisons in the network. It is evident when a network has a closed loop of direct and indirect evidence. Inconsistency occurs when there is a violation of this assumption due to discrepancy between this evidence. Following the global approach, we will compare model fit of consistency and inconsistency models using their DIC. Local approaches such as node-splitting will be considered in the presence of complex networks. We will assess reasons for inconsistency across the body of evidence using a meta-regression approach. Inconsistency assessments will be adapted accordingly to ensure that the assumption holds for CNMA.

Potential reporting bias such as publication bias and small study-effects will be assessed using a network funnel plot. A multivariate random effects meta-regression will be fitted to investigate whether heterogeneity may be further explained by the presence of effect modifiers. Where possible, subgroup analysis will be conducted. We will perform sensitivity analyses to evaluate the robustness of our findings. For example, use of different priors in the NMA will be examined as results are suggested to be sensitive to the chosen priors in presence of small amount of data, or the influence of different studies (small population or low-quality studies) on our results will be examined.

### Realist synthesis

To complement and explain the results of the NMA, we will employ a realist approach [41] to synthesise a broad and diverse body of literature (i.e., quantitative, qualitative and mixed-methods studies) in the form of a programme theory regarding complex causal mechanisms and how these interact with individuals’ agency and social context to produce outcomes. In particular, we will follow the five key stages for realist reviews, as described by Pawson et al. [41] and captured in the RAMESES quality and publication guidelines [42]: (1) locate existing theories, (2) search for evidence, (3) study selection, (4) extract and organise data and (5) synthesise the evidence and draw conclusions.

#### Phase 1: Locating existing theories

We will start by conducting initial scoping searches of key literature to identify available theories that may explain how lifestyle modification interventions for adults with learning disabilities work, for whom they work and why they may work in particular circumstances and not in others. This exploratory searching differs from the more systematic literature searching that will be conducted in phase 2, as the objective here will be to quickly locate the range of possible theories that may be relevant. Identified theories will then be synthesised into an initial programme theory that will be discussed with our advisory and PPI groups and further refined based on their input.

#### Phase 2: Searching for evidence

After running preliminary scoping searches and developing a ‘rough’ programme theory, we will use the results of the literature search previously described. This will be used to identify a relevant body of literature that might contain data with which to further develop and refine our programme theory. Given that the goal of a realist synthesis is to make sense of diverse evidence about complex interventions applied in different settings, we will seek to include (published and unpublished) studies of any methodological design, such as RCTs, controlled studies, uncontrolled studies, surveys, and qualitative studies of participants’ views and experiences of interventions. Specifically, the realist review is likely to include evidence covering the following:

- Studies focusing on adults with learning disabilities (as previously defined) and reporting any intervention designed to change the following unhealthy lifestyle behaviours: smoking, alcohol use, unhealthy diet, physical inactivity and/or sedentary behaviour
- Studies reporting barriers and facilitators to the implementation and uptake of lifestyle modification interventions in adults with learning disabilities
- Studies that could provide opportunities for transferable learning (e.g., studies reporting interventions for adults with learning disabilities, but not targeting the above-mentioned lifestyle behaviours)

#### Phase 3: Study selection

The key consideration for selecting studies to be included in realist reviews is the extent to which these include data that can contribute to programme theory development. Therefore, standard quality appraisal checklists, which cannot capture issues like the conceptual richness of a study or its relevance to the review question, have been argued to be insufficient for realist reviews [41]. Similar to previously published work [43, 44], we will assess potentially eligible studies based on their relevance to programme theory development (high/low) and, for those articles classified as ‘highly relevant’, we will also assess rigour and trustworthiness of findings. For instance, if an article reports the results of a qualitative process evaluation of an intervention conducted in the UK, it will be judged as ‘highly relevant’. However, if the study was based on a very small sample size, which did not allow for data saturation (i.e., further data collection might have yielded substantive new information), then the methodological rigour of the work will be scored as ‘low’.

To ensure consistency in the assessments of rigour, quality appraisal tools will be used. For intervention study designs, the aforementioned ROB version 2 and ROBINS-I will be used. Qualitative papers will be appraised using the Critical Appraisal Skills Programme (CASP) Qualitative Studies checklist [45]. If studies include quantitative methodology, the quantitative checklist of The Standard Quality Assessment Criteria for Evaluating Primary Research Papers will be used [46] as it can be applied to multiple study designs. All screening and selection decisions will be made by a single reviewer, with a 10% random sub-sample of citations reviewed independently by a second, to ensure consistency in decisions.

#### Phase 4: Extracting and organising data

Main study characteristics (e.g., objectives, sample, study design, risk behaviour targeted) will be extracted into an Excel spreadsheet by one reviewer and checked for accuracy by a second. Full texts of included papers will be uploaded into NVivo QRS International (a qualitative data management software) and verbatim sections of text, namely those understood as contexts, mechanisms and their relationships to outcomes, will be coded. It is anticipated that quantitative data will be mostly used to shed light on the outcome patterns (e.g., changes in blood glucose levels and body weight), whereas qualitative data will provide a more in-depth understanding of contexts and mechanisms. We will start the coding and analysis process by using a set of ‘key’ papers, namely studies that are likely to have a major contribution to the development of our programme theory due to their high relevance and conceptual richness. The resulting coding framework will then be applied to the rest of the papers, moving from the ones that are more relevant and specific to our programme theory to those that are less relevant and specific.

#### Phase 5: Synthesising the evidence and drawing conclusions

In the final phase of the review, we will continue to use a realist logic of analysis to build context–mechanism–outcome configurations (CMOCs), while searching to identify relationships not just within the same articles, but across sources. For instance, we will seek to explain how and why context may have influenced observed outcomes, by comparing interventions that have been successful in changing unhealthy lifestyle behaviours among adults with learning disabilities against those that have not. Such analysis will allow us to understand the behaviour of the most important mechanisms under different contexts and to build more transferable CMOCs. We will work with our steering/advisory and PPI groups to finalise our evidence informed framework of what works, for whom and in what contexts in relation to lifestyle behaviour modification interventions for adults with learning disabilities and produce a set of actionable recommendations to inform policy and practice.

### Logic model

To bring together the findings from the two syntheses (NMA and realist review) in a meaningful way, we will develop a logic model. A logic model is a summary diagram which maps out the underpinning pathway and causal mechanism of how complex interventions work [47]. Logic models have predominantly been used in programme evaluations; however, more recently their importance to contribute to synthesising findings from systematic reviews has been recognised [48]. Our approach will combine data from both quantitative and qualitative designs, treated as textual (qualitative) data. The process involves charting, categorising the data, and thematic synthesis methods to develop a process-orientated logic model [49]. The logic model aims to portray how interventions operate. We will present our logic model to our PPI members and will ask them to provide their input on the relevance of the findings for adults with learning disabilities, how might the information help them improve these unhealthy behaviours in practice, to identify any gaps in the evidence, and what they think we should be doing to make it easier for them to improve these lifestyle behaviours. This is an essential step to inform the development of lifestyle modification interventions to be delivered in the NHS and social care services and improve the health of adults with learning disabilities.

## Discussion

Our study employs a mixed-methods approach to meet the objectives mentioned above. The NMA and CNMA will allow us to assess the relative effectiveness of different lifestyle modification interventions and the components that contribute to improvements in modifiable health risk factors, respectively. The realist synthesis will complement and explain the results of the NMA and CNMA and generate an in-depth understanding of the interventions. Lastly, the logic model will inform the pathway and causal mechanism associated with these complex interventions. To our knowledge, this protocol describes the first study of its kind.

We foresee some limitations while undertaking this study. Inconsistent reporting of intervention detail may be a challenge while coding theories or BCT. This will also influence the reporting of intervention components and the ability to run a NMA on all outcomes described. Diverse outcome measurements may limit our capacity to combine results from different studies in NMA. In case of CNMA, should there be a lack of studies comparing various combinations of components, it would impede our ability to disentangle the relative effects of the various components. It may also lead the model to have insufficient power, which would have to be overcome by grouping components together in a clinically meaningful manner. We anticipate some practical issues, such as those related to the update of PRISMA statement to the new PRISMA 2020 statement [50] and contacting authors of old, hard-to-obtain papers. However, we will follow the Open Science practices during the conduct of our study. Our protocol has been pre-registered in the PROSPERO database where any important protocol amendments or updates will be recorded. We plan to publish our search syntax, extracted data and analytical codes as supplementary material upon completion and publication of our study.

In conclusion, the paucity of population-specific lifestyle interventions contributes to the challenges of behaviour change in adults with learning disabilities. This study will provide an evidence-base from which various stakeholders can develop effective interventions for adults with learning disabilities. The evidence will also help prioritise and inform research recommendations for future primary research so that people with learning disabilities live happier, healthier and longer lives.

## Supplementary Information

**Additional file 1:** PRISMA-P 2015 Checklist. Description of data: This file contains preferred reporting items for systematic review and meta-analysis protocols (PRISMA-P) 2015 checklist for this protocol.

**Additional file 2:** MEDLINE search strategy. Description of data: This file contains the search strategy in MEDLINE database.

## Data Availability

The datasets used and/or analysed during the current study are available from the corresponding author on reasonable request.

## Abbreviations

ASSIA: Applied Social Sciences Index and Abstracts
BCT: Behaviour change technique
CENTRAL: Cochrane Central Register of Controlled Trials
CINAHL: Cumulative Index to Nursing and Allied Health Literature
CMOC: Context–mechanism–outcome configurations
CNMA: Component network meta-analysis
EDD: Energy deficit diet
DIC: Deviance information criteria
EPPI-Centre: Evidence for Policy and Practice Information and Co-ordinating Centre
GRADE: Grading of Recommendations Assessment, Development and Evaluation
ISPOR: International Society for Pharmacoeconomics Outcomes Research
ISRCTN: International Standard Randomised Controlled Trials Number
NMA: Network meta-analysis
OpenSIGLE: Open Systems for Information on Grey Literature in Europe
OR: Odds ratio
PPI: Patient and Public Involvement
PRISMA-P: Preferred Reporting Items for Systematic Reviews and Meta-Analyses Protocols
RCT: Randomised controlled trials
ROBINS: Risk of Bias In Non-randomised Studies of Interventions
SMD: Standardised mean differences
TCS: Theory Coding Scheme
