# Supplementary material for: Understanding the effectiveness and underlying mechanisms of lifestyle modification interventions in adults with learning disabilities: protocol for a mixed-methods systematic review
Source: Syst Rev. 2021 Sep 20;10:251. doi: 10.1186/s13643-021-01808-0 (PMC8453997; doi:10.1186/s13643-021-01808-0)
Supplement: Supplementary file 2 — Additional file 2: MEDLINE search strategy. Description of data: This file contains the search strategy in MEDLINE database. [file 13643_2021_1808_MOESM2_ESM.docx]

**MEDLINE Search Strategy**

| **Condition** | |
| --- | --- |
| 1 | ((development* or learn*) adj2 disorder*).tw. |
| 2 | exp intellectual disability/ |
| 3 | ((learn* or development* or mental* or intellect* or cognitv*) adj2 (deficien* or disab*or disorder* or deficien* or difficult* or impair* or handicap* or retard* or sub?normal* or challenge*)).tw. |
| 4 | (cretin* or feeble minded* or imbecil* or moron*).tw. |
| **Health risk behaviours** | |
| 5 | exp smoking/ or exp cigarette smoking/ |
| 6 | ((smok* adj2 (behavio?r or habit* or us* or consum*)) or (tobacco or cigarette)).tw. |
| 7 | exp binge drinking/ or exp alcohol consumption/ |
| 8 | ((alcohol or ethanol or drink*) adj2 (problem* or harm* or hazard* or depend* or binge or us* or consum* or misuse* or behavio?r or habit*)).tw. |
| 9 | (unhealth* adj2 (food or diet*) adj2 (habit* or consum*)).tw. |
| 10 | exp sedentary time/ or exp sedentary lifestyle/ |
| 11 | ((sedentary or passive or inactive or physical*) adj2 (life?style* or behavio?r* or liv* or li?e or time)).tw. |
| 12 | exp obesity/ |
| 13 | ((over or excess) adj2 weight).tw. |
| **Interventions and expected outcomes** | |
| 14 | exp behavior therapy/ or exp cognitive behavioral therapy/ or exp psychotherapy/ or exp family therapy/ or exp counseling/ |
| 15 | ((life?style* or behavio?r*) adj2 (modif* or interven* or change* or program*)).tw. |
| 16 | ((behavio?r* or cogniti* or CBT or psycho?therap* or psycho?educat or psycho?social or counsel*) adj2 (session* or therap* or technique* or modif* or interven* or change*)).tw. |
| 17 | (health* adj2 (promot* or educat* or life?style*)).tw. |
| 18 | exp health promotion/ or exp health education/ |
| 19 | exp smoking cessation/ |
| 20 | ((tobacco or smok* or nicotine or replace* or relapse) adj2 (cessat* or stop or reduc* or prevent* or therap*)).tw. |
| 21 | exp diet therapy/ or exp caloric restriction/ or exp low fat diet/ or exp low carbohydrate diet/ or exp portion size/ or exp nutritional support/ |
| 22 | (health* adj2 (diet* or weight)).tw. |
| 23 | ((calorie* or portion* or serv* or size*) adj2 (control* or reduc* or restrict*)).tw. |
| 24 | ((diet* or nutri* or food or carb* or protein* or fat*) adj2 (educat* or guide* or habit* or intake)).tw. |
| 25 | exp physical activity/ or exp exercise/ |
| 26 | (interven* adj2 (physic* or exercise*)).tw. |
| 27 | ((moderat* or vigo?r*) adj2 (activit* or exercise* or train*)).tw. |
| 28 | ((exercise* or physic*) adj2 (aerobic* or train* or fit* or active* or endur*)).tw. |
| 29 | ((gym* or circuit* or aqua* or walk* or jog* or run* or swim* or weight* lift* or (strength or resist* or circuit* or aerobic*)) adj2 train*).tw. |
| 30 | ((fat or body or weight) adj2 loss).tw. |
| 31 | ((health or weight or obes*) adj2 (loss or reduc* or manage*)).tw. |
| **Grouped terms** | |
| 32 | or/1-4 |
| 33 | or/5-13 |
| 34 | or/14-31 |
| 35 | 32 and 33 and 34 |
| 36 | limit 35 to humans |

.
